# Supplementary material for: Amino Acid-Based Boron Carriers in Boron Neutron Capture Therapy (BNCT)
Source: Pharmaceutics. 2023 Nov 23;15(12):2663. doi: 10.3390/pharmaceutics15122663 (PMC10748186; doi:10.3390/pharmaceutics15122663)
Supplement: Supplementary file 1 [file pharmaceutics-15-02663-s001.zip › pharmaceutics-2703699-SI.pdf]

Amino acid-based boron carriers in Boron Neutron Capture Therapy (BNCT)

Juulia Järvinen [1], Herkko Pulkkinen [2], Jarkko Rautio [1] and Juri M. Timonen [1,3,*]

[1] School of Pharmacy, Faculty of Health Sciences, University of Eastern Finland
[2] Department of Technical Physics, Faculty of Natural Sciences and Forestry, University of Eastern Finland
[3] Drug Research Program, Division of Pharmaceutical Chemistry and Technology, Faculty of Pharmacy, University of Helsinki, Viikinkaari 5E, P.O. Box 56, 00014 Helsinki, Finland
* Correspondence: juri.timonen@helsinki.fi

Table of Contents

Table S1. *In vitro* boron accumulation and distribution studies on melanoma models using amino acid-based boron carriers........ 2

Table S2. *In vitro* boron accumulation and distribution studies of brain tumor models using amino acid-based boron carriers........ 3

Table S3. *In vitro* boron accumulation and distribution studies on miscellaneous cell lines using amino acid-based boron carriers........ 6

Table S4. Biodistribution and PET studies on *in vivo* melanoma models using amino acid-based boron carriers........ 9

Table S5. Biodistribution and neutron capture studies on *in vivo* brain tumor models using amino acid-based boron carriers........ 10

Table S6. Miscellaneous *In vivo* studies using amino acid-based boron carriers. 10

Table S7. *In vitro* neutron capture survival studies on cancer cell lines using amino acid-based boron carriers........ 11

Table S8. *In vivo* neutron capture studies using amino acid-based boron carriers. 11

Table S9. *In vitro* toxicity studies of amino acid-based boron carriers. 12

REFERENCES 13

Table S1. *In vitro* boron accumulation and distribution studies on melanoma models using amino acid-based boron carriers.

#	Cell line	Comp.	Conc.	Incubation time	Measured boron accumulation	Ref.
1	B16	L-BPA	2mM	24 h	1.5±0.4 μgB/10^7cells [a,b]	[1]
		**13**			2.2±0.4 μgB/10^7cells [a,b]	
		**14**			3.1±0.5 μgB/10^7cells [a,b]	
		**15**			2.3±0.6 μgB/10^7cells [a,b]	
2		BPA*HCl	2mM	3 h	1.3±0.1 μgB/10^7cells [a,c]	[2]
				12 h	2.0±0.1 μgB/10^7cells [a,c]	
				24 h	2.4±0.1 μgB/10^7cells [a,c]	
		**23**	2mM	3 h	0.5±0.2 μgB/10^7cells [a,c]	
				12 h	1.9±0.2 μgB/10^7cells [a,c]	
				24 h	2.0±0.1 μgB/10^7cells [a,c]	
		**24**	2mM	3 h	0.5±0.2 μgB/10^7cells [a,c]	
				12 h	1.9±0.2 μgB/10^7cells [a,c]	
				24 h	1.8±0.1 μgB/10^7cells [a,c]	
		**25**	2mM	3 h	0.6±0.2 μgB/10^7cells [a,c]	
				12 h	1.1±0.1 μgB/10^7cells [a,c]	
				24 h	1.4±0.2 μgB/10^7cells [a,c]	
3		*cis*-**29**	50 ppm of boron	2.5 h	distribution study [d]	[3]
		trans-**29**				
4	B16F10	BPA*HCl	1 mg/mL ≈ 4.1 mM	1 h	0.2±0.07 μgB/10^7cells [a,c]	[4]
				3 h	0.6±0.06 μgB/10^7cells [a,c]	
				24 h	1.6±0.3 μgB/10^7cells [a,c]	
		BPA	0.1 mM[e]	1 min	0.03±0.01 % dose/mg prot. ≈ 0.3±0.1 ngB/mg prot. [b,f]	[5]
				5 min	0.62±0.01 % dose/mg prot. ≈ 6±0.1 ngB/mg prot. [b,f]	
				30 min	0.57±0.06 % dose/mg prot. ≈ 6±0.6 ngB/mg prot. [b,f]	
5		**3**	0.1 mM[e]	1 min	0.23±0.03 % dose/mg prot. ≈ 2±0.3 ngB/mg prot. [b,f]	
				5 min	0.59±0.1 % dose/mg prot. ≈ 6±1 ngB/mg prot. [b,f]	
				30 min	0.80±0.07 % dose/mg prot. ≈ 8±0.7 ngB/mg prot. [b,f]	
6		**10**	1 mM	1 h	390±3 μgB/10^7cells [a,b]	[6]
			2 mM		700±3 μgB/10^7cells [a,b]	
			5 mM		1040±6 μgB/10^7cells [a,b]	
			10 mM		1280±12 μgB/10^7cells [a,b]	
6	MK-T	BPA	0.45mM	3 h	76±4 ngB/mg prot. [a,g]	[7]
				5 h	64±11 ngB/mg prot. [a,g]	
				10 h	37±15 ngB/mg prot. [a,g]	
				24 h	27±9 ngB/mg prot. [a,g]	
				32 h	86±7 ngB/mg prot. [a,g]	
				48 h	110±12 ngB/mg prot. [a,g]	
7	A1059	BPA*HCl	1 mg/mL ≈ 4.1 mM	1 h	0.1±0.03 μgB/10^7cells [a,c]	[4]
				3 h	0.3±0.03 μgB/10^7cells [a,c]	

				24 h	0.8±0.05 μgB/10^{7}cells [a,c]	
8	TA1059-1	BPA*HCl	1 mg/mL ≈ 4.1 mM	1 h	0.3±0.07 μgB/10^{7}cells [a,c]	
				3 h	0.6±0.09 μgB/10^{7}cells [a,c]	
				24 h	2.0±0.2 μgB/10^{7}cells [a,c]	
9	Ihara	**5**	2 mM	24 h	Not quantified [h]	[8]
		**6**				
		**7**				
		**8**				
10	B16F1	[^{18}F]-**9**	370 kBq	15 min	5.77±0.21 %AR/10^{5}cells [i]	[9]
				30 min	6.92±0.24 %AR/10^{5}cells [i]	
				60 min	8.37±0.46 %AR/10^{5}cells [i]	
				120 min	7.39±1.24 %AR/10^{5}cells [i]	
				240 min	7.26±0.54 %AR/10^{5}cells [i]	
11	MRA 27	**11**	100 μg B/mL ≈ 1 mM	3h	3.32±0.61 μgB/10^{7}cells [j,k]	[10]
12	SK-23 Mel	BPA-F	0.05mM	2 h	17.1±2.0 μgB/L [a,b,l,m]	[11]
				6 h	14.0±1.7 μgB/L [a,b,l,n]	
		**47**	0.05mM	2 h	15.5±1.1 μgB/L [a,b,l,m]	
				6 h	7.5±1.4 μgB/L [a,b,l,n]	
		**48**	0.05mM	2 h	11.6±1.4 μgB/L [a,b,l,m]	
				6 h	8.9±1.4 μgB/L [a,b,l,n]	

[a]estimated graphically from image of the original publication, [b] measured with Inductively Coupled Plasma Optical Emission Spectroscopy (ICP-OES), [c]measured with Inductively Coupled Plasma Atom Emission Spectroscopy (ICP-AES), [d]measured with Secondary Ion Mass Spectrometry (SIMS) imaging, [e]calculated based on the original paper as follows: concentration of BPA or **3** in solution of 0.5 mg of BPA-fructose mixture with molar ratio 1:2.6 in 1 mL is 1.0 mmol/L, and adding 100 μL into 900 μL results in concentration of 0.1 mM, [f]calculated based on the dose calculated as described above and result normalization described in the original paper as follows: "The accumulation rate was calculated as % dose/mg protein from the quantified dose of boron in added 3-BPA and 4-BPA, respectively (n = 3)" values are from measurements without LAT1-inhibitor, [g]measured with Inductively Coupled Plasma Mass Spectrometry (ICP-MS), [h]measured with Fluorine Nuclear Magnetic Resonance Spectroscopy (F-NMR), [i]measured with gamma counter, uptake expressed as percent of the applied radioactivity used in the assay vial relative to untreated control, [j]measured with Direct-current Plasma Atom Emission Spectrometry (DCP-AES), [k]calculated from data of the original publication, [l]measured from filtrate after scrapping cells from Petri dish into 3 mL of water and rinsing the Petri dish with additional 2 mL of water, [m]blank control: 10.0±1.5 μgB/L [a,b,l], [n]blank control: 5.1±1.6 μgB/L [a,b,l]

Table S2. *In vitro* boron accumulation and distribution studies of brain tumor models using amino acid-based boron carriers

#	Cell line	Comp.	Conc.	Incubation time	Measured boron accumulation	Ref.
1	C6	[^{10}B] -BPA	1mM	24 h	0.2 μgB/10^{7}cells [a,b,c]	[12]
		**5**			0.1 μgB/10^{7}cells [a,b,c]	
		**7**			0.1 μgB/10^{7}cells [a,b,c]	
2		L-BPA	2mM	24 h	1.4±0.08 μgB/10^{7}cells [a,d]	[1]
		**13**			1.4±0.3 μgB/10^{7}cells [a,d]	
		**14**			1.7±0.06 μgB/10^{7}cells [a,d]	
		**15**			1.9±0.1 μgB/10^{7}cells [a,d]	
3		L-BPA	2mM	24 h	0.72±0.02 μgB/10^{7}cells [a,d]	[13]
		**13**			0.96±0.7 μgB/10^{7}cells [a,d]	
		cis-**40**			1.57±0.16 μgB/10^{7}cells [a,d]	
		trans-**40**			2.23±0.14 μgB/10^{7}cells [a,d]	
4	U87-MG	[^{14}C]-L-BPA	18.5kBq	5 min	1.21±0.21 %AD/10^5 cells [a,e]	[14]

		$[^{18}F]$-**9**	185 KBq	5 min	1.16±0.16 %AD/10^5 cells [a,f]	
5		BPA	0.05mM	3 min	76±<4 ppm ^{10}B [a,g]	[15]
				1 h	20±<4 ppm ^{10}B [a,g]	
				2 h	30±11 ppm ^{10}B [a,g]	
				4 h	22±<4 ppm ^{10}B [a,g]	
		**41**		3 min	20±<4 ppm ^{10}B [a,g]	
				1 h	17±<4 ppm ^{10}B [a,g]	
				2 h	23±<4 ppm ^{10}B [a,g]	
				4 h	33±16 ppm ^{10}B [a,g]	
		**42**		3 min	22±<4 ppm ^{10}B [a,g]	
				1 h	20±<4 ppm ^{10}B [a,g]	
				2 h	30±12 ppm ^{10}B [a,g]	
				4 h	24±12 ppm ^{10}B [a,g]	
		**43**		3 min	22±9 ppm ^{10}B [a,g]	
				1 h	11±<4 ppm [a,g]	
				2 h	23±6 ppm ^{10}B [a,g]	
				4 h	16±<4 ppm ^{10}B [a,g]	
		**44**		3 min	23±<4 ppm ^{10}B [a,g]	
				1 h	79±22 ppm ^{10}B [a,g]	
				2 h	171±16 ppm ^{10}B [a,g]	
				4 h	208±38 ppm ^{10}B [a,g]	
		**45**		3 min	60±9 ppm ^{10}B [a,g]	
				1 h	135±40 ppm ^{10}B [a,g]	
				2 h	314±80 ppm ^{10}B [a,g]	
				4 h	299±67 ppm ^{10}B [a,g]	
		**46**		3 min	28±<4ppm ^{10}B [a,g]	
				1 h	148±27 ppm ^{10}B [a,g]	
				2 h	346±42 ppm ^{10}B [a,g]	
				4 h	300±43 ppm ^{10}B [a,g]	
6		BPA	0.1 mM[h]	1 min	0.13±0.02 % dose/mg prot. ≈ ±0.1 ngB/mg prot. [g,i]	[5]
				5 min	0.20±0.02 % dose/mg prot. ≈ 2±0.2 ngB/mg prot. [g,i]	
				30 min	0.48±0.03 % dose/mg prot. ≈ 5±0.3 ngB/mg prot. [g,i]	
		**3**	0.1 mM[h]	1 min	0.33±0.04 % dose/mg prot. ≈ 3±0.4 ngB/mg prot. [g,i]	
				5 min	0.28±0.04 % dose/mg prot. ≈ 3±0.4 ngB/mg prot. [g,i]	
				30 min	0.45±0.05 % dose/mg prot. ≈ 5±0.5 ngB/mg prot. [g,i]	
7	LN-229	$[^{14}C]$-L-BPA	18.5 kBq	5 min	6.27±0.67 %AD/10^5 cells [a,e]	[14]
		$[^{18}F]$-**9**	185 KBq		5.74±0.48 %AD/10^5 cells [a,f]	
8		BPA	0.05mM	3 min	48±8 ppm ^{10}B [a,g]	[15]
				1 h	55±<4 ppm ^{10}B [a,g]	
				2 h	24±8 ppm ^{10}B [a,g]	
				4 h	32±<4 ppm ^{10}B [a,g]	

		**41**		3 min	38±27 ppm ^{10}B [a,g]	
				1 h	38±8 ppm ^{10}B [a,g]	
				2 h	29±<4 ppm ^{10}B [a,g]	
				4 h	24±<4 ppm ^{10}B [a,g]	
		**42**		3 min	17±7 ppm ^{10}B [a,g]	
				1 h	42±13 ppm ^{10}B [a,g]	
				2 h	24±9 ppm ^{10}B [a,g]	
				4 h	27±<4 ppm ^{10}B [a,g]	
		**43**		3 min	8±<4 ppm ^{10}B [a,g]	
				1 h	27±<4 ppm ^{10}B [a,g]	
				2 h	20±10 ppm ^{10}B [a,g]	
				4 h	16±<4 ppm ^{10}B [a,g]	
		**44**		3 min	47±28 ppm ^{10}B [a,g]	
				1 h	142±54 ppm ^{10}B [a,g]	
				2 h	190±10 ppm ^{10}B [a,g]	
				4 h	212±16 ppm ^{10}B [a,g]	
		**45**		3 min	206±68 ppm ^{10}B [a,g]	
				1 h	459±100 ppm ^{10}B [a,g]	
				2 h	277±39 ppm ^{10}B [a,g]	
				4 h	210±30 ppm ^{10}B [a,g]	
		**46**		3 min	27±<4 ppm ^{10}B [a,g]	
				1 h	59±33 ppm ^{10}B [a,g]	
				2 h	57±42 ppm ^{10}B [a,g]	
				4 h	109±21 ppm ^{10}B [a,g]	
9	F98	[^{10}B]-L-BPA	1mM	24 h	3.27 ± 0.3 µg ^{10}B/10^7 cells [b]	[13]
		rac-**40**			1.97 ± 0.2 µg ^{10}B/10^7 cells [b]	[16]
10	T98G	[^{10}B]-L-BPA	110 µg/mL B equivalent ≈ 1 mM[j]	1 h	421 ± 127 µg ^{10}B/g [k,l]	[17]
				2 h	437 ± 126 µg ^{10}B/g [k,l]	
				6 h	777 ± 337 µg ^{10}B/g [k,l]	
				8 h	830 ± 162 µg ^{10}B/g [k,l]	
				16 h	784 ± 218 µg ^{10}B/g [k,l]	
11	LN-18	[^{14}C]-L-BPA	18.5 kBq	5 min	2.10±0.48 %AD/10^5 cells [a,e]	[14]
		**[^{18}F]9**	185 KBq		1.84±0.32 %AD/10^5 cells [a,f]	
12	U118 MG	[^{14}C]-L-BPA	18.5 kBq	5 min	4.48±0.65 %AD/10^5 cells [a,e]	[14]
		**[^{18}F]9**	185 KBq		4.94±0.42 %AD/10^5 cells [a,f]	
13	U-251 MG	[^{14}C]-L-BPA	18.5 kBq	5 min	1.98±0.46 %AD/10^5 cells [a,e]	[14]
		**[^{18}F]9**	185 KBq		1.61±0.16 %AD/10^5 cells [a,f]	
14	U343	**19**	50 µgB/mL ≈ 0.46 mM	30 min	26±<3 µgB/g [a,b,m]	[18]
				1 h	33±3.5 µgB/g [a,b,m]	
				2 h	47±4.9 µgB/g [a,b,m]	
				4 h	56±5.8 µgB/g [a,b,m]	
				8 h	71±7.4 µgB/g [a,b,m]	
				20 h	88±9.1 µgB/g [a,b,m]	
				48 h	88±9.3 µgB/g [a,b,m]	
			9 µgB/mL ≈ 0.08 mM	20 h	13±92.2 µgB/g [a,b,m]	

			23 μgB /mL ≈ 0.21 mM		32±10 μgB/g [a,b,m]	
			47 μgB /mL ≈ 0.43 mM		70±11 μgB/g [a,b,m]	
15	G98T	[^{10}B]-L-BPA-F	110 μg/mL B equivalent ≈ 1 mM[j]	2 h	711 ± 93 μg ^{10}B/g [k,n]	[19]
				6 h	1112 ± 231 μg ^{10}B/g [k,n]	
		cis-**29**		1 h	217 ± 47 μg B/g [k,n]	
				2 h	240 ± 66 μg B/g [k,n]	
				4 h	360 ± 77 μg B/g [k,n]	
				6 h	377 ± 84 μg B/g [k,n]	
16	A172	L-BPA	2mM	24 h	2.79±0.07 μgB/10^7cells [a,d]	[13]
		**13**			2.95±0.02 μgB/10^7cells [a,d]	
		cis-**40**			3.01±0.08 μgB/10^7cells [a,d]	
		trans-**40**			3.87±0.04 μgB/10^7cells [a,d]	
17	U81	L-BPA	2mM	24 h	0.690.17 μgB/10^7cells [a,d]	[13]
		**13**			0.84±0.39 μgB/10^7cells [a,d]	
		cis-**40**			1.39±0.2 μgB/10^7cells [a,d]	
		trans-**40**			1.58±0.43 μgB/10^7cells [a,d]	
18	U-251	L-BPA	2mM	24 h	1.1±0.2 μgB/10^7cells [a,d]	[13]
		**13**			0.53±0.22 μgB/10^7cells [a,d]	
		cis-**40**			0.50±0.14 μgB/10^7cells [a,d]	
		trans-**40**			0.53±0.22 μgB/10^7cells [a,d]	

[a]estimated graphically from image of the original publication, [b]measured with Inductively Coupled Plasma Atom Emission Spectroscopy (ICP-AES), [c]no error bars raported in original data, [d]measured with Inductively Coupled Plasma Optical Emission Spectroscopy (ICP-OES), [e]percentage of the administered dose per 10^5 cells, measured with scintillation counter, [f]percentage of the adminis tered dose per 10^5 cells, measured with gamma counter, [g]measured with Inductively Coupled Plasma Mass Spectrometry (ICP-MS), [h]calculated based on the original paper as follows: concentration of BPA or **3** in solution of 0.5 mg of BPA-fructose mixture with molar ratio 1:2.6 in 1 mL is 1.0 mmol/L, and adding 100 μL into 900 μL results in concentration of 0.1 mM, [i]calculated based on the dose calculated as described above and result normalization described in the original paper as follows: “The accumulation rate was calculated as % dose/mg protein from the quantified dose of boron in added 3-BPA and 4-BPA, respectively (n = 3)” values are from measurements without LAT1-inhibitor, [j]concentration described in the original paper as follows: “110 μg/ml boron equivalent of ^{10}BPA”, [k]measured using Secondary Ion Mass Spectrometry Imaging (SIMS), [l] μg of ^{10}B/g (wet weight) summed up from results of three different cell compartments (nucleus, mitochondria-rich perinuclear cytoplasm and remaining cytoplasm), [m] μg of B/g of cells, [n] μg of ^{10}B/g (wet weight) summed up from results of three different cell compartments (nucleus, mitochondria-rich perinuclear cytoplasm and remaining cytoplasm)

Table S3. *In vitro* boron accumulation and distribution studies on miscellaneous cell lines using amino acid-based boron carriers.

#	Cancer type	Cell line	Compound	Conc.	Incubation time	Measured boron accumulation	Ref.
1	Squamous carcinoma	KB	^{10}B-BPA	1 mM	24 h	0.70 μgB/10^7cells [a,b,c]	[12]
			**5**			0.70 μgB/10^7cells [a,b,c]	
			**7**			0.75 μgB/10^7cells [a,b,c]	
2		FaDu	[^{14}C]-L-BPA	18.5 kBq	5 min	4.63±0.19 %AD/10^5 cells [a,d]	[14]
			**[^{18}F]9**	185 KBq		4.65±0.39 %AD/10^5 cells [a,e]	
3		SAS	L-BPA	2 mM	24 h	2.5±0.7 μgB/10^7cells [a,f]	[1]
			**13**			2.0±0.3 μgB/10^7cells [a,f]	
			**14**			1.6±0.4 μgB/10^7cells [a,f]	

			**15**			2.5±0.7 μgB/10^7cells [a,f]	
4	Human epithelioma	HeLa	^{10}B-BPA	1 mM	24 h	0.70 μgB/10^7cells [a,b,c]	[12]
			**5**			0.65 μgB/10^7cells [a,b,c]	
			**7**			0.60 μgB/10^7cells [a,b,c]	
5	Epidermal carcinoma	A-253	[^{14}C]-L-BPA	18.5 kBq	5 min	5.10±0.19 %AD/10^5 cells [a,d]	[14]
			[^{18}F]-**9**	185 KBq		7.23±0.68 %AD/10^5 cells [a,e]	
6	Hepatocellular carcinoma	HuH-7	[^{18}F]-**9**	370 kBq	15 min	1.12±0.11 %AR/10^5cells [g]	[9]
					30 min	1.29±0.05 %AR/10^5cells [g]	
					60 min	1.61±0.09 %AR/10^5cells [g]	
					120 min	1.22±0.13 %AR/10^5cells [g]	
					240 min	1.10±0.08 %AR/10^5cells [g]	
7	Colorectal adenocarcinoma	CaCo-2	[^{18}F]-**9**	370 kBq	15 min	2.98±0.30 %AR/10^5cells [g]	[9]
					30 min	3.49±0.20 %AR/10^5cells [g]	
					60 min	5.24±0.20 %AR/10^5cells [g]	
					120 min	4.77±0.19 %AR/10^5cells [g]	
					240 min	4.87±0.21 %AR/10^5cells [g]	
8	Healthy tissue	TIG	BPA*HCl,	2mM	3 h	1.1±0.1 μgB/10^7cells [a,b]	[2]
					12 h	1.0±0.1 μgB/10^7cells [a,b]	
					24 h	1.5±<0.1 μgB/10^7cells [a,b]	
			**23**	2mM	3 h	0.3±<0.1 μgB/10^7cells [a,b]	
					12 h	0.5±0.2 μgB/10^7cells [a,b]	
					24 h	1.0±0.2 μgB/10^7cells [a,b]	
			**24**	2mM	3 h	0.3±<0.1 μgB/10^7cells [a,b]	
					12 h	0.5±<0.1 μgB/10^7cells [a,b]	
					24 h	0.9±<0.1 μgB/10^7cells [a,b]	
			**25**	2mM	3 h	0.2±<0.1 μgB/10^7cells [a,b]	
					12 h	0.3±<0.1 μgB/10^7cells [a,b]	
					24 h	0.6±0.2 μgB/10^7cells [a,b]	
9	Healthy tissue	3T3	BPA	0.05 mM	3 min	32±5 ppm ^{10}B [a,g]	[15]
					1 h	25±8 ppm ^{10}B [a,g]	
					2 h	41±13 ppm ^{10}B [a,g]	
					4 h	70±20 ppm ^{10}B [a,g]	
			**41**	0.05 mM	3 min	21±<5 ppm ^{10}B [a,g]	
					1 h	22±7 ppm ^{10}B [a,g]	
					2 h	18±10 ppm ^{10}B [a,g]	
					4 h	35±8 ppm ^{10}B [a,g]	
			**42**	0.05 mM	3 min	24±6 ppm ^{10}B [a,g]	
					1 h	22±10 ppm ^{10}B [a,g]	
					2 h	30±13 ppm ^{10}B [a,g]	
					4 h	54±16 ppm ^{10}B [a,g]	
			**43**	0.05 mM	3 min	13±<5 ppm ^{10}B [a,g]	
					1 h	20±<5 ppm ^{10}B [a,g]	
					2 h	15±7 ppm ^{10}B [a,g]	
					4 h	54±18 ppm ^{10}B [a,g]	
			**44**	0.05 mM	3 min	10±<5 ppm ^{10}B [a,g]	

					1 h	42±5 ppm ^{10}B [a,g]	
					2 h	75±19 ppm ^{10}B [a,g]	
					4 h	40±24 ppm ^{10}B [a,g]	
			**45**	0.05 mM	3 min	19±<5 ppm ^{10}B [a,g]	
					1 h	48±4 ppm ^{10}B [a,g]	
					2 h	146±58 ppm ^{10}B [a,g]	
					4 h	96±13 ppm ^{10}B [a,g]	
			**46**	0.05 mM	3 min	18±<5 ppm ^{10}B [a,g]	
					1 h	58±23 ppm ^{10}B [a,g]	
					2 h	75±9 ppm ^{10}B [a,g]	
					4 h	146±13 ppm ^{10}B [a,g]	
10	Breast cancer	MCF-7	BPA-F	0.05 mM	2 h	13.2±1.0 μgB/L[a,f,h,j]	[11]
					6 h	15.4±0.5 μgB/L[a,f,h,j]	
			**47**	0.05 mM	2 h	6.8±4.3 μgB/L[a,f,h,j]	
					6 h	7.3±4.4 μgB/L[a,f,h,j]	
			**48**	0.05 mM	2 h	4.4±3.1 μgB/L[a,f,h,j]	
					6 h	4.9±2.1 μgB/L[a,f,h,j]	
11	Human pancreatic adenocarcinoma	T3M-4	BPA	0.1 mM[h]	1 min	0.82±0.11 % dose/mg prot. ≈ 8±1.1 ngB/mg prot. [g,i]	[5]
					5 min	3.28±0.39 % dose/mg prot. ≈ 33±3.9 ngB/mg prot. [g,i]	
					30 min	5.40±0.45 % dose/mg prot. ≈ 54±4.5 ngB/mg prot. [g,i]	
			**3**	0.1 mM[h]	1 min	0.78±0.09 % dose/mg prot. ≈ 8±0.9 ngB/mg prot. [g,i]	
					5 min	3.55±0.40 % dose/mg prot. ≈ 36±4.0 ngB/mg prot. [g,i]	
					30 min	5.62±0.63 % dose/mg prot. ≈ 56±6.3 ngB/mg prot. [g,i]	
12	Human lung carcinoma	A549	BPA	0.1 mM[h]	1 min	0.14±0.04 % dose/mg prot. ≈ 1±0.4 ngB/mg prot. [g,i]	[5]
					5 min	0.66±0.08 % dose/mg prot. ≈ 7±0.8 ngB/mg prot. [g,i]	
					30 min	3.92±0.20 % dose/mg prot. ≈ 39±2.0 ngB/mg prot. [g,i]	
			**3**	0.1 mM[h]	1 min	0.48±0.12 % dose/mg prot. ≈ 5±1.2 ngB/mg prot. [g,i]	
					5 min	1.22±0.13 % dose/mg prot. ≈ 12±1.3 ngB/mg prot. [g,i]	
					30 min	4.02±0.24 % dose/mg prot. ≈ 40±2.4 ngB/mg prot. [g,i]	

[a]estimated graphically from image of the original publication, [b]measured with Inductively Coupled Plasma Atom Emission Spectroscopy (ICP-AES), [c]no error bars raported in original data, [d]percentage of the administered dose per 10^5 cells, measured with scintillation counter, [e]percentage of the administered dose per 10^5 cells, measured with gamma counter, [f]measured with Inductively Coupled Plasma Optical Emission Spectroscopy (ICP-OES), [g]measured with gamma counter, uptake expressed as percent of the applied radioactivity used in the assay vial relative to untreated control, [h]measured from filtrate after scrapping cells from Petri dish into 3 mL of water and rinsing the Petri dish with additional 2 mL of water, [i]blank control: 5.9±3.9 μgB/L[a,f,h], [j]blank control: 7.9±3.2 μgB/L[a,f,h]

Table S4. Biodistribution and PET studies on *in vivo* melanoma models using amino acid-based boron carriers.

#	Animal & Cancer model	Compound (administr.)	Study type	Analysis method	Ref.
1	Greenes melanoma transplanted in the anterior chamber or as a sub choroidal transplant in the posterior chamber on rabbit	750 mg/kg of BPA orally as one dose	Biodistribution, neutron irradiation	Radiography	[20]
2	Greene's melanoma s.c. on Syrian (golden) hamster	100 mg/kg inj i.p. of BPA-F, **3-F, 4-F**	Biodistribution	ICP-AES	[21]
3	B16F1 or B16F10 cells transplanted s.c. on C57BL/6 male mice	1 mCi inj. i.v. of [^{18}F]**9**	Biodistribution	Autoradiograph	[22]
4	B16-F10 cells implanted s.c. into the right shoulder of C57BL/6 female mice	11.1 MBq inj. iv. for PET and 2.1 MBq for biodistribution study of [^{18}F]**10**	PET & Biodistribution	PET and ICP-OES	[6]
5	Harding-Passey melanoma implanted i.m. on BALB/c mice	62.5 µg B/g b.w. inj. i.p. of BPA or **11**	Biodistribution		[10]
6	Harding-Passey murine melanoma implanted s.c. on BALB/c female mice	3, 6, 12 mg inj. i.p. of DL-BPA**,** L-[^{10}B]BPA, and D-[^{10}B]BPA	Biodistribution	Prompt-Gamma Boron Analysis, Neutron capture radiography	[23]
7	B16BL6 melanoma implanted on B6D2F$_1$ female mice	800-1000 ppm B inj. i.v. of 30**-35**	Biodistribution	ICP-AES	[24]
8	B16 cells injected s.c. on female BALB/c mice	24 mg B/kg b.w. inj. i.p. of BPA, *cis*-**30**, *trans*-**30**	biodistribution	ICP-OES	[3]
9	B16 cells implanted s.c. in syngeneic female C57BL/6 mice	24 mg B/kg b.w. of BPA-F, DL-*cis*-**29**-F, DL-*trans*-**29**-F, DL-*cis*-**30**-F, DL-*trans*-**30**-F *i*nj. ip.	bio- and micro distribution	ICP-OES, SIMS	[25]
10	B16F10 cells s.c. in right hind legs of BALB/c mice	1 mg of **3**-F, BPA-F	biodistribution	ICP-MS	[5]

Table S5. Biodistribution and neutron capture studies on *in vivo* brain tumor models using amino acid-based boron carriers.

#	Tumor model	Compounds, dose and administration	Study type	Analysis method	Ref.
1	Fischer rats implanted with F98 glioma cells into the right caudate nucleus intracerebral implantation,	30 mg B/kg b.w. inj. i.v. or i.c. of [^{10}B]BPA	Biodistribution	DCP-AES	[26]
2	U-87 MG brain tumor on female BALB/c and SCID mice	150 KBq in 100 µL of [^{18}F]**9**.	Biodistribution	gamma counter	[14]
3	LN-229 brain tumor on female BALB/c and SCID mice	150 KBq in 100 µL of [^{18}F]**9**.	Biodistribution	gamma counter	[14]
4	U-118 MG brain tumor on female BALB/c and SCID mice	150 KBq in 100 µL of [^{18}F]**9**.	Biodistribution	gamma counter	[14]
5	U-251 MG brain tumor on female BALB/c and SCID mice	150 KBq in 100 µL of [^{18}F]**9** a.	Biodistribution	gamma counter	[14]
6	F98 cells implanted intracerebrally into the caudate nucleus of Fischer rats.	31 mg B/kg b.w. inj. i.p. **11**	Biodistribution	DCP-AES	[10]
7	F98 cells implanted in the brain of syngeneic Fischer rats	12 mg B/kg b.w. inj. i.p. of BPA-F or 16 mg B/kg of DL-*cis*-**29**	Bio- and microdistribution	ICP-OES, SIMS	[25]
8	F98 cells implanted in the brain of syngeneic Fischer rats	16 mg B/kg b.w. inj. i.p. of *cis*-**29**	Bio- and microdistribution	ICP-OES, SIMS	[27]
9	F98 cells implanted in the brain on male Fischer 344 rats	12 mg B/kg b.w. inj. i.v. of L-BPA, *cis*-**40,** *trans*-**40**	Biodidistribution	ICP-AES,	[16]

Table S6. Miscellaneous *In vivo* studies using amino acid-based boron carriers.

#	Cell type	Animal & Cancer model	Compound(s), dose and administration	Study type	Analysis method	Ref.
1	Healthy	Male ddYY mice	3.5 MBg inj. i.v. of [^{18}F]**9**	Biodistribution	Not reported	[28]
2	Epidermal carcinoma	A253 on female BALB/c and SCID mice	150 KBq in 100 µL of [^{18}F]**9**	Biodistribution	gamma counter	[14]

3	Squamous cell carcinoma	FaDu on female BALB/c and SCID mice	150 KBq in 100 µL of [^{18}F]**9**	Biodistribution	gamma counter	[14]
4	Carcinoma	FM3A transplanted s.c. on C3H/He male mice	1 mCi i.v. inj tail vein of [^{18}F]**9**	Biodistribution	Autoradiography	[22]

Table S7. *In vitro* neutron capture survival studies on cancer cell lines using amino acid-based boron carriers.

#	Cancer type	Cell line	Compound(s)	Concentration	Incubation time	Neutron source	Ref
1	Melanoma	Mel-J	BPA	10 ppm ^{10}B	2 h	RA-3 Nuclear Reactor, CNEA, Buenos Aires	[29]
2	Melanoma	M8	BPA	10 ppm ^{10}B	2 h	RA-3 Nuclear Reactor, CNEA, Buenos Aires, Argentina	[29]
3	Melanoma	B16	L-BPA, **13**,**14**,**15**	2mM	24 h	Kyoto University Research Reactor	[1]
4	Glioblastoma	C6	L-BPA, **13**,**14**,**15**	2mM	24 h	Kyoto University Research Reactor	[1]
5	Squamous carcinoma	SAS	L-BPA, **13**,**14**,**15**	2mM	24 h	Kyoto University Research Reactor	[1]
6	Glioma	U87	L-BPA, **20**	1µM to 1mM	30 to 180 min	Albany-SUNY Dynamitron accelerator.	[30]

Table S8. *In vivo* neutron capture studies using amino acid-based boron carriers.

#	Tumor model	Compounds, dose and administration	Analysis method	Ref.
1	Greenes melanoma transplanted in the anterior chamber or as a sub choroidal transplant in the posterior chamber on rabbit	750 mg/kg of BPA orally as one dose	Radiography	[20]
2	B16-F10 cells implanted s.c. into the right shoulder of C57BL/6 female mice	40 mg inj. i.v. of **10**	tumor volume, body weight	[6]
3	A1059 or TA1059-1 injected s.c. in buttock on Male C57BL/6 mice	400 mg/kg inj. i.p. inj. of [^{10}B]BPA	tumor volume	[4]
4	Fischer rats implanted with F98 glioma cells into the right caudate nucleus intracerebral implantation,	30 mg B/kg b.w. inj. i.c. of [^{10}B]BPA		[26]

5	F98 cells implanted in the brain on male Fischer 344 rats	12 mg B/kg b.w. inj. i.v. of L-BPA, *cis*-**40**, *trans*-**40**	survival time	[16]

Table S9. *In vitro* toxicity studies of amino acid-based boron carriers.

#	Compound	Cell line	Conc.	toxicity	Ref
1	L-BPA	B16		IC_{50} > 2 mM	[1]
			15 mM	survival 20±1% after 3 days	[2]
		C6		IC_{50} > 2 mM	[1]
				IC_{50} > 10 mM	[13]
		SAS		IC_{50} > 2 mM	[1]
		U81		IC_{50} > 10 mM	[13]
		A172		IC_{50} > 10 mM	
		U87[a]	0.1 mM	67.0±2.0% viability	[15]
		LN229[b]	0.1 mM	79.6±3.6% viability	
		3T3[c]	0.1 mM	53.0±1.6% viability	
2	**5**	C6	1-20 mM	no toxic found	[11]
3	**10**	B16-F10	50 mM	no toxicity founs	[6]
4	**11**	MRA 27		IC_{50} ≈ 65 μg/mL ≈ 28 mM	[31]
5	**13**	B16		IC_{50} = 5.6 mM	[1]
		C6		IC_{50} = 6.6 mM	
		SAS		IC_{50} = 7.8 mM	
		U251		IC_{50} = 8.8 mM	[13]
		A172		IC_{50} = 5.55 mM	
6	**14**	B16		IC_{50} = 6.6 mM	[1]
		C6		IC_{50} = 2.4 mM	
		SAS		IC_{50} = 4.9 mM	
7	**15**	B16		IC_{50} = 5.1 mM	
		C6		IC_{50} = 4.7 mM	
		SAS		IC_{50} = 5.6 mM	
8	**18**	MRA 27		IC_{50} ≈ 97 μg/mL ≈ 29 mM	[31]
9	**19**	U343	0-0.5n mM	no toxicity found	[18]
10	**23**	B16	15 mM	survival 60±2% after 3 days	[2]
		TIG	15 mM	survival 66±2% after 3 days	
11	**24**	B16	15 mM	survival 60±2% after 3 days	
		TIG	15 mM	survival 71±2% after 3 days	
12	**25**	B16	15 mM	survival 62±2% after 3 days	
		TIG	15 mM	survival 73±2% after 3 days	
13	*cis*-**40**	C6		IC_{50} = 2.5 mM	[13]
		A172		IC_{50} = 2.0 mM	
		U251		IC_{50} > 10 mM	
14	*trans*-**40**	C6		IC_{50} = 2.1 mM	[13]
		A172		IC_{50} = 1.9 mM	
		U251		IC_{50} > 10 mM	

15	**41**	U87	0.1 mM	61.3±5.0% viability	[15]
		LN229	0.1 mM	57.2±6.2% viability	
		3T3	0.1 mM	109.9 ± 1.1% viability	
16	**42**	U87	0.1 mM	66.2±3.2% viability	
		LN229	0.1 mM	69.9±2.9% viability	
		3T3	0.1 mM	42.9±0.7% viability	
17	**43**	U87	0.1 mM	65.8±3.3% viability	
		LN229	0.1 mM	67.3±1.9% viability	
		3T3	0.1 mM	47.6 ± 1.5% viability	
18	**44**	U87	0.1 mM	60.1±1.9% viability	
		LN229	0.1 mM	53.2±2.0% viability	
		3T3	0.1 mM	63.6±0.9% viability	
19	**45**	U87	0.1 mM	67.4±1.6% viability	
		LN229	0.1 mM	60.7±5.2% viability	
		3T3	0.1 mM	60.2±2.3% viability	
20	**46**	U87	0.1 mM	70.2±0.1% viability	
		LN229	0.1 mM	70.8±1.4% viability	
		3T3	0.1 mM	66.6±1.8% viability	
21	**47**	SK-23 Mel	0-0.1 mM	< 5% toxicity	[11]
		MCF-7	0-0.1 mM	< 5% toxicity	

[a]the used 1% DMSO showed 97% viability, [b]the used 1% DMSO showed 91% viability, [c]the used 1% DMSO showed 57% viability

REFERENCES

1. Hattori, Y.; Kusaka, S.; Mukumoto, M.; Uehara, K.; Asano, T.; Suzuki, M.; Masunaga, S.; Ono, K.; Tanimori, S.; Kirihata, M. Biological Evaluation of Dodecaborate-Containing L-Amino Acids for Boron Neutron Capture Therapy. *J. Med. Chem.* **2012**, *10*, 6980–6984.

2. Nemoto, H.; Cai, J.; Asao, N.; Iwamoto, S.; Yamamoto, Y. Synthesis and Biological Properties of Water-Soluble p-Boronophenylalanine Derivatives. Relationship between Water Solubility, Cytotoxicity, and Cellular Uptake. *J. Med. Chem.* **1995**, *38*, 1673–1678, doi:10.1021/jm00010a012.

3. Kabalka, G.W.; Shaikh, A.L.; Barth, R.F.; Huo, T.; Yang, W.; Gordnier, P.M.; Chandra, S. Boronated unnatural cyclic amino acids as potential delivery agents for neutron capture therapy. *Appl. Radiat. Isot.* **2011**, *69*, 1778–1781, doi:10.1016/j.apradiso.2011.03.035.

4. Tsuboi, T.; Kondoh, H.; Hiratsuka, J.; Mishima, Y. Enhanced Melanogenesis Induced by Tyrosinase Gene-Transfer Increases Boron-Uptake and Killing Effect of Boron Neutron Capture Therapy for Amelanotic Melanoma. *Pigment Cell Res.* **1998**, *11*, 275–282, doi:10.1111/j.1600-0749.1998.tb00736.x.

5. Kondo, N.; Hirano, F.; Temma, T. Evaluation of 3-Borono-L-Phenylalanine as a Water-Soluble Boron Neutron Capture Therapy Agent. *Pharmaceutics* **2022**, *14*, doi:10.3390/pharmaceutics14051106.

6. Li, J.; Shi, Y.; Zhang, Z.; Liu, H.; Lang, L.; Liu, T.; Chen, X.; Liu, Z. A Metabolically Stable Boron-Derived Tyrosine Serves as a Theranostic Agent for Positron Emission Tomography Guided Boron Neutron Capture Therapy. *Bioconjug. Chem.* **2019**, *30*, 2870–2878, doi:10.1021/acs.bioconjchem.9b00578.

7. Belkhou, R.; Abbé, J.C.; Pham, P.; Jasner, N.; Sahel, J.; Dreyfus, H.; Moutaouakkil, M.; Massarelli, R. Uptake and metabolism of boronophenylalanine in human uveal melanoma cells in culture

Relevance to boron neutron capture therapy of cancer cells. *Amino Acids* **1995**, *8*, 217–229, doi:10.1007/BF00806495.

8. Hattori, Y.; Yamamoto, H.; Ando, H.; Kondoh, H.; Asano, T.; Kirihata, M.; Yamaguchi, Y.; Wakamiya, T. Synthesis and evaluation as MRI probe of the trifluoromethylated p-boronophenylalanine and its alcohol derivative. *Bioorganic Med. Chem.* **2007**, *15*, 2198–2205, doi:10.1016/j.bmc.2006.12.043.

9. Wingelhofer, B.; Kreis, K.; Mairinger, S.; Muchitsch, V.; Stanek, J.; Wanek, T.; Langer, O.; Kuntner, C. Preloading with L-BPA, L-tyrosine and L-DOPA enhances the uptake of [18F]FBPA in human and mouse tumour cell lines. *Appl. Radiat. Isot.* **2016**, *118*, 67–72, doi:10.1016/j.apradiso.2016.08.026.

10. Yong, J.-H.; Barth, R.F.; Wyzlic, I.M.; Soloway, A.H.; Rotaru, J.H. In vitro and in vivo evaluation of o-Carboranylalanine as a potential boron delivery agent for neutron capture therapy. *Anticancer Res.* **1995**, *16*, 113–120.

11. Bonjoch, J.; Drew, M.G.B.; González, A.; Greco, F.; Jawaid, S.; Osborn, H.M.I.; Williams, N.A.O.; Yaqoob, P. Synthesis and evaluation of novel boron-containing complexes of potential use for the selective treatment of malignant melanoma. *J. Med. Chem.* **2008**, *51*, 6604–6608, doi:10.1021/jm8007745.

12. Hattori, Y.; Asano, T.; Niki, Y.; Kondoh, H.; Kirihata, M.; Yamaguchi, Y.; Wakamiya, T. Study on the compounds containing 19F and 10B atoms in a single molecule for the application to MRI and BNCT. *Bioorganic Med. Chem.* **2006**, *14*, 3258–3262, doi:10.1016/j.bmc.2005.10.062.

13. Hattori, Y.; Kusaka, S.; Mukumoto, M.; Ishimura, M.; Ohta, Y.; Takenaka, H.; Uehara, K.; Asano, T.; Suzuki, M.; Masunaga, S.I.; et al. Synthesis and in vitro evaluation of thiododecaborated α, α-Cycloalkylamino acids for the treatment of malignant brain tumors by boron neutron capture therapy. *Amino Acids* **2014**, *46*, 2715–2720, doi:10.1007/s00726-014-1829-5.

14. Yoshimoto, M.; Honda, N.; Kurihara, H.; Hiroi, K.; Nakamura, S.; Ito, M.; Shikano, N.; Itami, J.; Fujii, H. Non-invasive estimation of 10B-4-borono-L-phenylalanine-derived boron concentration in tumors by PET using 4-borono-2-18F-fluoro-phenylalanine. *Cancer Sci.* **2018**, *109*, 1617–1626, doi:10.1111/cas.13553.

15. Chio, C.M.; Huang, Y.C.; Chou, Y.C.; Hsu, F.C.; Lai, Y.B.; Yu, C.S. Boron Accumulation in Brain Tumor Cells through Boc-Protected Tryptophan as a Carrier for Boron Neutron Capture Therapy. *ACS Med. Chem. Lett.* **2020**, *11*, 589–596, doi:10.1021/acsmedchemlett.0c00064.

16. Futamura, G.; Kawabata, S.; Nonoguchi, N.; Hiramatsu, R.; Toho, T.; Tanaka, H.; Masunaga, S.I.; Hattori, Y.; Kirihata, M.; Ono, K.; et al. Evaluation of a novel sodium borocaptate-containing unnatural amino acid as a boron delivery agent for neutron capture therapy of the F98 rat glioma. *Radiat. Oncol.* **2017**, *12*, 1–11, doi:10.1186/s13014-017-0765-4.

17. Chandra, S.; Lorey, D.R. SIMS ion microscopy imaging of boronophenylalanine (BPA) and 13C15N-labeled phenylalanine in human glioblastoma cells: Relevance of subcellular scale observations to BPA-mediated boron neutron capture therapy of cancer. *Int. J. Mass Spectrom.* **2007**, *260*, 90–101, doi:10.1016/j.ijms.2006.09.006.

18. Olsson, P.; Black, M.; Capala, J.; Coderre, J.; Hartman, T.; Makar, M.; Malmquist, J.; Pettersson, J.; Tilly, N.; Sjoberg, S.; et al. Uptake, toxicity and radiation effects of the boron compounds DAAC-1 and DAC-1 in cultured human glioma cells. *Int. J. Radiat. Oncol. Biol.* **1998**, *73*.

19. Chandra, S.; Ahmad, T.; Barth, R.F.; Kabalka, G.W. Quantitative evaluation of boron neutron capture therapy (BNCT) drugs for boron delivery and retention at subcellular-scale resolution in human glioblastoma cells with imaging secondary ion mass spectrometry (SIMS). *J. Microsc.* **2014**, *254*, 146–156, doi:10.1111/jmi.12126.

20. Packer, S.; Coderre, J.; Saraf, S.; Fairchild, R.; Hansrote, J.; Perry, H. Boron neutron capture therapy of anterior chamber melanoma with p- boronophenylalanine. *Investig. Ophthalmol. Vis. Sci.* **1992**, *33*, 395–403.

21. Hiratsuka, J.; Yoshino, K.; Kondoh, H.; Imajo, Y.; Mishima, Y. Biodistribution of boron concentration on melanoma-bearing hamsters after administration of p-, m-, o-boronophenylalanine. *Japanese J. Cancer Res.* **2000**, *91*, 446–450, doi:10.1111/j.1349-7006.2000.tb00965.x.

22. Kubota, R.; Yamada, S.; Ishiwata, K.; Tada, M.; Ido, T.; Kubota, K. Cellular accumulation of 18f-labelled boronophenylalanine depending on DNA synthesis and melanin incorporation: A double-tracer microautoradiographic study of B16 melanomas in vivo. *Br. J. Cancer* **1993**, *67*, 701–705, doi:10.1038/bjc.1993.129.

23. Coderre, J.A.; Glass, J.D.; Fairchild, R.G.; Roy, U.; Cohen, S.; Fand, I. Selective Targeting of Boronophenylalanine to Melanoma in BALB/c Mice for Neutron Capture Therapy. *Cancer Res.* **1987**, *47*, 6377–6383.

24. Kabalka, G.W.; Wu, Z.Z.; Yao, M.; Natarajan, N. The syntheses and in vivo biodistribution of novel boronated unnatural amino acids. **2004**, *61*, 1111–1115, doi:10.1016/j.apradiso.2004.05.012.

25. Barth, R.F.; Kabalka, G.W.; Yang, W.; Huo, T.; Nakkula, R.J.; Shaikh, A.L.; Haider, S.A.; Chandra, S. Evaluation of unnatural cyclic amino acids as boron delivery agents for treatment of melanomas and gliomas. *Appl. Radiat. Isot.* **2014**, *88*, 38–42, doi:10.1016/j.apradiso.2013.11.133.

26. Barth, R.F.; Yang, W.; Rotaru, J.H.; Moeschberger, M.L.; Joel, D.D.; Nawrocky, M.M.; Goodman, J.H.; Soloway, A.H. Boron neutron capture therapy of brain tumors: Enhanced survival following intracarotid injection of either sodium borocaptate or boronophenylalanine with or without blood-brain barrier disruption. *Cancer Res.* **1997**, *57*, 1129–1136, doi:10.1016/S0360-3016(00)00421-1.

27. Chandra, S.; Barth, R.F.; Haider, S.A.; Yang, W.; Huo, T.; Shaikh, A.L.; Kabalka, G.W. Biodistribution and Subcellular Localization of an Unnatural Boron-Containing Amino Acid (Cis-ABCPC) by Imaging Secondary Ion Mass Spectrometry for Neutron Capture Therapy of Melanomas and Gliomas. *PLoS One* **2013**, *8*, doi:10.1371/journal.pone.0075377.

28. Ishiwata, K.; Ido, T.; Mejia, A.A.; Ichihashi, M.; Mishima, Y. Synthesis and radiation dosimetry of 4-borono-2-[18F]fluoro-d,l-phenylalanine: A target compound for PET and boron neutron capture therapy. *Int. J. Radiat. Appl. Instrumentation. Part* **1991**, *42*, 325–328, doi:10.1016/0883-2889(91)90133-L.

29. Rossini, A.E.; Dagrosa, M.A.; Portu, A.; Saint Martin, G.; Thorp, S.; Casal, M.; Navarro, A.; Juvenal, G.J.; Pisarev, M.A. Assessment of biological effectiveness of boron neutron capture therapy in primary and metastatic melanoma cell lines. *Int. J. Radiat. Biol.* **2015**, *91*, 81–89, doi:10.3109/09553002.2014.942013.

30. He, T.; Musah, R.A. Evaluation of the Potential of 2-Amino-3-(1,7-dicarba- closo-dodecaboranyl-1-thio)propanoic acid as a boron neutron capture therapy agent. *ACS Omega* **2019**, *4*, 3820–3826, doi:10.1021/acsomega.8b03407.

31. Yong, J.H.; Barth, R.F.; Rotaru, J.H.; Wyzlic, I.M.; Soloway, A.H. Evaluation of in vitro cytotoxicity of carboranyl amino acids, their chemical precursors and nido carboranyl amino acids for Boron Neutron Capture Therapy. *Anticancer Res.* **1995**, *15*, 2039–2043.
